# Post-Treatment HIV-1 Controllers with a Long-Term Virological Remission after the Interruption of Early Initiated Antiretroviral Therapy ANRS VISCONTI Study

**DOI:** 10.1371/journal.ppat.1003211

**Published:** 2013-03-14

**Authors:** Asier Sáez-Cirión, Charline Bacchus, Laurent Hocqueloux, Véronique Avettand-Fenoel, Isabelle Girault, Camille Lecuroux, Valerie Potard, Pierre Versmisse, Adeline Melard, Thierry Prazuck, Benjamin Descours, Julien Guergnon, Jean-Paul Viard, Faroudy Boufassa, Olivier Lambotte, Cécile Goujard, Laurence Meyer, Dominique Costagliola, Alain Venet, Gianfranco Pancino, Brigitte Autran, Christine Rouzioux, the ANRS VISCONTI Study Group

**Affiliations:** 1 Institut Pasteur, Unité de Régulation des Infections Rétrovirales, Paris, France; 2 Université Pierre et Marie Curie, INSERM UMR-S 945 Immunité et Infection, Hôpital Pitié-Salpêtrière, Paris, France; 3 Centre Hospitalier Régional d'Orléans, Service des Maladies Infectieuses et Tropicales, Orléans, France; 4 AP-HP, CHU Necker-Enfants Malades, Laboratoire de Virologie, Paris, France; 5 EA 3620, Université Paris-Descartes, Sorbonne Paris Cité, Paris, France; 6 INSERM U1012, Université Paris-Sud 11, Le Kremlin Bicêtre, France; 7 UPMC Univ Paris 06, UMR_S 943, Paris, France; 8 INSERM, U943, Paris, France; 9 AP-HP, Hôtel-Dieu, Paris, France; 10 INSERM U1018, Université Paris-Sud 11, Le Kremlin Bicêtre, France; 11 AP-HP, Hôpital de Bicêtre, Service de Médecine Interne, Le Kremlin Bicêtre, France; 12 AP-HP, Hôpital de Bicêtre, Département d'épidémiologie, Le Kremlin Bicêtre, France; 13 AP-HP, Groupe hospitalier Pitié-Salpétrière, Service de Maladies Infectieuses et Tropicales, Paris, France; SAIC-Frederick, United States of America

## Abstract

Combination antiretroviral therapy (cART) reduces HIV-associated morbidities and mortalities but cannot cure the infection. Given the difficulty of eradicating HIV-1, a functional cure for HIV-infected patients appears to be a more reachable short-term goal. We identified 14 HIV patients (post-treatment controllers [PTCs]) whose viremia remained controlled for several years after the interruption of prolonged cART initiated during the primary infection. Most PTCs lacked the protective HLA B alleles that are overrepresented in spontaneous HIV controllers (HICs); instead, they carried risk-associated HLA alleles that were largely absent among the HICs. Accordingly, the PTCs had poorer CD8+ T cell responses and more severe primary infections than the HICs did. Moreover, the incidence of viral control after the interruption of early antiretroviral therapy was higher among the PTCs than has been reported for spontaneous control. Off therapy, the PTCs were able to maintain and, in some cases, further reduce an extremely low viral reservoir. We found that long-lived HIV-infected CD4+ T cells contributed poorly to the total resting HIV reservoir in the PTCs because of a low rate of infection of naïve T cells and a skewed distribution of resting memory CD4+ T cell subsets. Our results show that early and prolonged cART may allow some individuals with a rather unfavorable background to achieve long-term infection control and may have important implications in the search for a functional HIV cure.

## Author Summary

There is a renewed scientific interest in developing strategies allowing long-term remission in HIV-1-infected individuals. Very rare (<1%) patients are able to spontaneously control viremia to undetectable levels (HIV controllers, HICs). However, the possibility to translate their mechanisms of control to other patients is uncertain. Starting antiretroviral therapy during primary infection may provide significant benefits to HIV-infected patients (i.e. reduction of viral reservoirs, preservation of immune responses, protection from chronic immune activation). Indeed, we have observed that some HIV-infected patients interrupting a prolonged antiretroviral therapy initiated close to primary infection are able to control viremia afterwards. We present here 14 of such post-treatment controllers (PTCs). We show that PTCs have achieved control of infection through mechanisms that are, at least in part, different from those commonly observed in HICs and that their capacity to control is likely related to early therapeutic intervention. We found that PTCs were able, after therapy interruption, to keep, and in some cases further reduce, a weak viral reservoir. This might be related to the low contribution of long-lived cells to the HIV-reservoir in these patients. Finally, we estimated the probability of maintaining viral control at 24 months post-early treatment interruption to be ∼15%, which is much higher than the one expected for spontaneous control.

## Introduction

HIV-1 infection is normally characterized by sustained viral replication and a progressive loss of CD4+ T cells, leading to AIDS. Combined antiretroviral therapy (cART) suppresses viral replication and drastically reduces morbidity and mortality [1]. However, cART does not eradicate infected cells [2], and plasma viremia generally rebounds quickly after treatment is discontinued [3]. The existence of a few HIV-infected patients who spontaneously controlled HIV replication to undetectable levels for many years (HIV controllers [HICs]) suggests that a functional HIV cure or remission might be possible. However, how or whether other patients can achieve an HIC-like status is unclear.

Emerging evidence shows that early treatment has long-term benefits [4]. Treatment initiation during primary HIV-1 infection (PHI) rather than during chronic HIV-1 infection (CHI) may i) further reduce residual viral replication [5], ii) limit viral diversity [6] and viral reservoirs [7], iii) preserve innate immunity and T and B cell functions [8], [9], [10], and iv) accelerate immune restoration [11]. Most relevant studies show that CD4+ T cell counts are higher and that viral rebound occurs later (and at a lower level) after the discontinuation of treatment that began during PHI compared with treatment that began during CHI [12], [13]. Although in most cases, these advantages wane soon after treatment interruption [14], the existence of individuals in whom the viral load remains undetectable for several years after the interruption of prolonged therapy that was initiated very early after infection (post-treatment controllers [PTCs]) was reported by our group in 2010 [15]. These individuals hold important clues in the search for a functional HIV cure. Here, we have identified and characterized a group of 14 PTCs. We analyzed whether PTCs shared parameters that have been associated with spontaneous control of viremia in HICs, to explore whether the efficient control of infection in PTCs may indeed be derived from early treatment. In addition, we explored the level and distribution of the PTCs' latent viral reservoir in the blood. Indeed reaching functional cure will likely require reducing not only the size but also the distribution of the HIV reservoirs, particularly among the CD4 T cells with long lifespan or important clonogenic properties, as naïve and central-memory T cells (TCM).

## Results

### Study population

**Figure 1:**
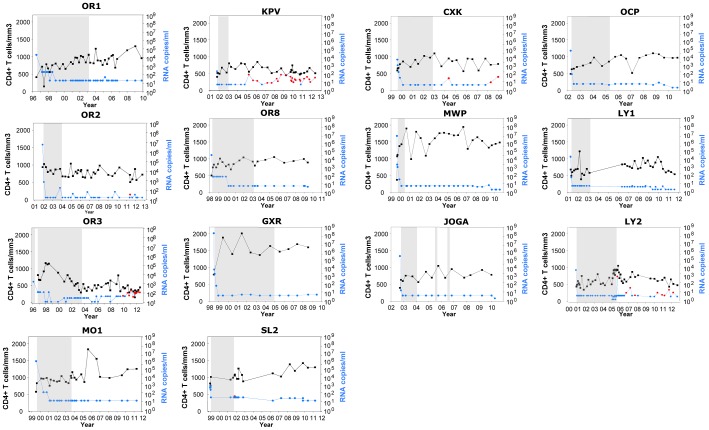
Long-term control of viremia and stable CD4+ T cell counts in fourteen patients after interruption of antiretroviral treatment initiated in primary HIV-1 infection. CD4+ T cell counts (in black) and plasma HIV-1 RNA viral loads (in blue) during the follow-up after PHI diagnosis in the 14 PTCs included in the study. The detectable viral loads after treatment interruption are indicated in red. The gray areas represent the periods during which the patients received cART.

**Figure 2:**
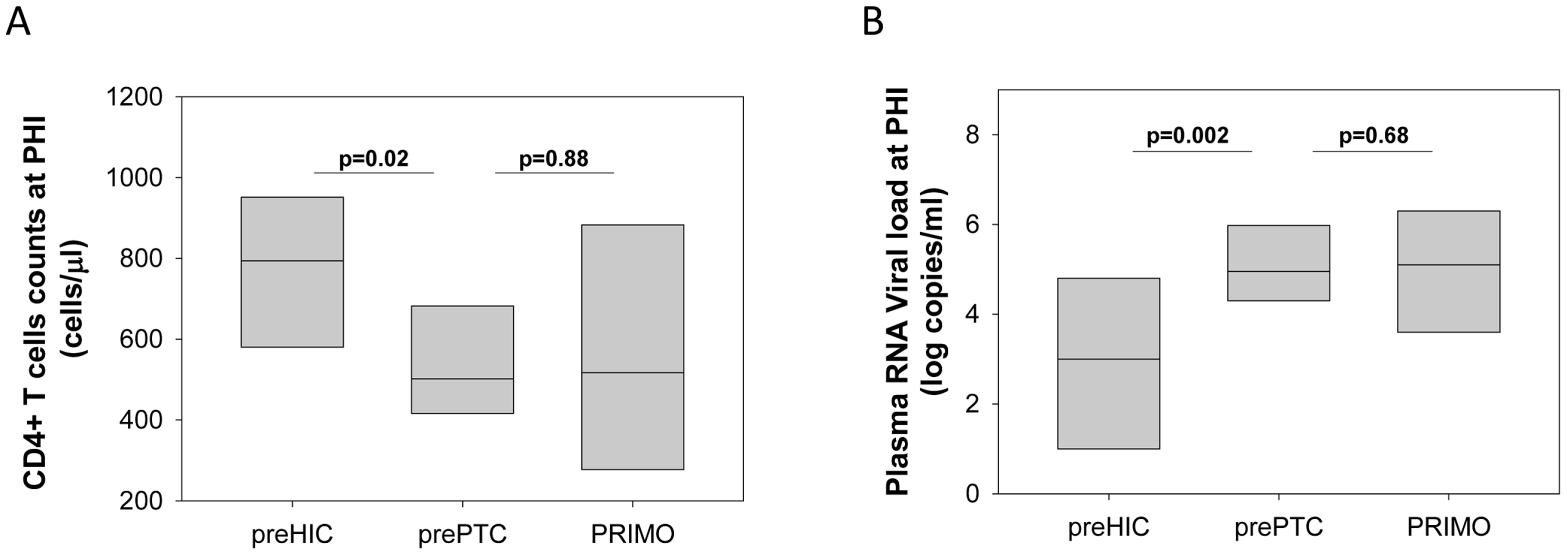
Patients to become post-treatment controllers have higher viral loads and lower CD4+ T cell counts than HIV controllers during primary HIV infection. CD4+ T cell counts (**A**) and plasma viral load (**B**) during the primary infection for the patients enrolled in the ANRS PRIMO cohort who later exhibited spontaneous control of infection (preHIC; n = 8) [16], for the PTCs included in our study (n = 14) and for the patients in the ANRS PRIMO cohort who did not control infection (n = 1,245). The median and the 10^th^ and 90^th^ percentiles are shown for each group.

We studied 14 HIV-1-infected patients with durable viral control following the interruption of effective cART that was initiated during PHI (PTCs). The patients' characteristics are reported in Table 1 and Figure 1. All 14 patients were diagnosed with PHI in the late 1990s or early 2000s. Twelve patients had a symptomatic primary infection. During PHI (1.6 [1.1–2.1] months estimated after initial exposure), the PTCs had higher viral loads (median 5.0 log HIV-1 RNA copies/ml) and lower CD4+ T cell counts (median 502 cells/µl; Table 1) compared with the 8 patients in the ANRS PRIMO cohort who subsequently exhibited spontaneous control of viremia (median 3.0 log HIV-1 RNA copies/ml of plasma and 794 CD4+ T cells/µl at PHI (2.2 [1.7–3.5] months estimated after exposure, p = 0.11 for the delay when compared to PTC) [16] (Figure 2). In contrast, PTC values during PHI were similar to those of patients in the ANRS PRIMO cohort who did not control their infection afterwards (5.1 log HIV RNA copies/ml and 517 cells/µl; Figure 2).

**Table 1:** Characteristics of PTC included in the study.

Code	Sex^1^	Year of diagnostic	PHI^2^	Fiebig ART initiation	ART combination^3^	Time on cART (months)	Time since interruption(months)	CD4 T cell counts (cells/µl)			Last HIV-1 DNA (copies/106 PBMC)	HIV-1 RNA VL^4^ (copies/ml plasma)		HIV-1 RNA VL since treatment interruption		
								First	cART discont.	Last		At PHI (Log)	Last during follow up	VL<50	VL>50<400	VL>400
**OR1**	M	1996	Sympt	V	2 NRTI	81	82	416	1057	959	134	4.3	<20	16/16		
**OR2**	F	2001	Sympt	V	3 NRTI+PI→3 NRTI	24	101	955	906	743	6	6.8	2	24/26	2/26	
**OR3**	F	1996	Sympt	I	2 NRTI→2 NRTI+PI	92	107	NA^5^	354	441	222	3.4^6^	91	18/28	10/28	
**OR8**	M	1998	Sympt	III	2 NRTI+PI→3 NRTI	60	72	502	915	886	122	5.0	<40	9/9		
**KPV**	M	2001	Sympt	V	NNRTI+2 NRTI→3NRTI	13	104	397	523	502	16	3.0	224	7/30	20/30	3/30
**GXR**	F	1998	Sympt	III	2 NRTI+PI	86	48	787	1636	1598	59	7.3	<40	5/5		
**CXK**	M	1999	Asympt	V	2 NRTI+PI	39	75	593	976	787	38	4.3	289	9/12	3/12	
**MWP**	M	1999	Sympt	V	2 NRTI+PI	12	115	371	1428	1400	120	7.1	1	21/21		
**JOGA**	F	2002	Sympt	IV	2 NRTI+PI^7^	17	72	393	734	779	8	5.9	<5	10/10		
**OCP**	M	2002	Sympt	V	2 NRTI+PI→3 NRTI	31	59	489	856	973	616	5.3	<20	11/11		
**LY1**	M	2001	Sympt	III	2 NRTI+PI→3 NRTI	23	101	682	833	541	36	4.9	<20	23/23		
**LY2**	M	2000	Asymp	V	3 NRTI	56	84	455	938	492	44	4.4	<40	13/22	8/22	1/22
**MO1**	M	1999	Sympt	V	2 NRTI+PI→2 NRTI+NNRTI	48	93	580	1044	1251	13	6.0	5	13/13		
**SL2**	M	1998	Sympt	V	3 NRTI+PI→3NRTI	34	113	822	993	1299	140	3.1	5	13/14	1/14	
**MEDIAN**		1999		V		36.5	89	502	927	837	51.5	5.0	<20			

1 M: Male, F: Female;

2 Primary HIV-1 infection, Symptomatic or Asymptomatic;

3 NRTI: Nucleoside Reverse Transcriptase Inhibitor, PI: Protease Inhibitor, NNRTI: Non-nucleoside Reverse Transcriptase Inhibitor;

4 VL: Viral load;

5 NA: non available;

6 First determination 4 days after initiating therapy;

7 Two transient treatments during pregnancies since first interruption.

The PTCs received standard cART (Table 1) available at the time, and their viral load became undetectable within a median of 3 months (0.5 to <8 months) after treatment began (Figure 1). The median cART duration was 36.5 months, and the plasma viral load was no longer detectable after the first undetectable sample during treatment. During the treatment period, all PTCs except two (OR2, with high CD4+ T cell counts of 955 cells/mm^3^ at PHI, and OR3, who was infected through a blood exposure accident and for whom no available CD4+ T cell counts were available before therapy) experienced an increase in their CD4+ T cell counts between PHI and treatment interruption (median 502 and 927 cells/mm^3^, respectively, p<0.001, n = 13). Following the interruption of cART, viral control persisted for a median of 89 months, and the CD4+ T cell counts remained stable (the median final CD4+ T cell count was 837 cells/mm^3^, p = 0.58, n = 14). Eight PTCs had viral loads below the detection limit in all available samples after treatment interruption, whereas occasional increases were recorded for the other six patients (Figure 1 and Table 1).

### The HIV-specific CD8+ T cell response of post-treatment HIV-1 controllers differs from that associated with HIV control in spontaneous HIV-1 controllers

**Figure 3:**
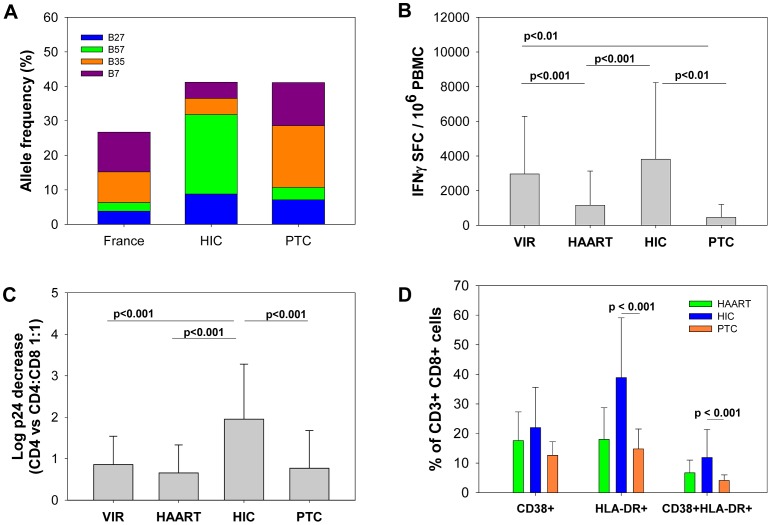
Post-treatment controllers differ from HIV controllers in terms of HLA class I profile, frequency and quality of the CD8+ T cell response and activation levels of CD8+ T cells. **A.** The frequencies of the protective alleles HLA-B*27 and B*57 and the risk alleles HLA-B*07 and B*35 in the general French population (n = 6094 alleles [20], www.allelefrequencies.net), HICs (n = 148 alleles) and PTCs (n = 28 alleles). The statistical analyses are shown in Table S2. **B.** The frequency of HIV-specific CD8+ T cells, estimated as the number of CD8+ T cells producing IFN-γ upon stimulation with optimal HIV-1 peptides (spot-forming cells, SFC) in untreated viremic patients (VIRs) (n = 57), treated patients (ARTs) (n = 60), HICs (n = 100) and PTCs (n = 12). **C.** The capacity of CD8+ T cells from VIRs (n = 22), ARTs (n = 14), HICs (n = 73) and PTCs (n = 14) to suppress the HIV-1 infection of autologous CD4+ T cells, as determined by the log-fold decrease in the level of secreted p24 (CD4 vs. CD4∶CD8 1∶1 cell cultures). **D.** The percentage of CD8+ T cells from ARTs (n = 5), HICs (n = 58) and PTCs (n = 8) that expressed CD38, HLA-DR or both CD38 and HLA-DR ex vivo. **B, C and D.** The mean and standard deviation for each group are shown.

We then compared specific parameters among the PTCs, HICs, patients with uncontrolled viremia (viremics [VIRs]) and patients receiving cART ([ARTs]; see methods). Protective HLA class I alleles (HLA-B*27 and B*57) have been consistently found to be overrepresented in cohorts of HICs [17], [18], [19] who spontaneously control HIV-1 infection. One of the PTCs had one HLA-B*57 allele and two PTCs had one HLA-B*27 allele. However, in contrast to the HICs from the ANRS HIV controller cohort, we found no overrepresentation of HLA-B*27 or HLA-B*57 in our PTC group compared with the general French population [20] (www.allelefrequencies.net; Figure 3A, Tables S1 and S2). Furthermore, the risk alleles HLA-B*07 and HLA-B*35 [17] were highly prevalent in the PTC group (29% of all HLA-B alleles), but they were underrepresented in the HIC group (p<0.001). Three and five of the 14 PTCs carried one HLA-B*07 allele and one HLA-B*35 allele, respectively. Three PTCs carried HLA-B*3501 (OR1, OR2 and OCP), whereas the other two (KPV and MWP) carried the HLA-B*3503 allele, which is associated with a more rapid progression to AIDS [21].

We and others have shown that most HICs have high frequencies of highly efficient HIV-1-specific CD8+ T cells [22], [23]. In fact, the elevated number of HIV-specific CD8+ T cells producing IFN-γ in the HICs was comparable to that in viremic patients (VIR) (Figure 3B). In contrast, we found that the PTCs had very weak HIV-specific CD8+ T cell responses during the viral control period. On average, the level of these responses in the PTCs were similar to that found in treated patients (ART), during both PHI and CHI (not shown), and were much lower than in viremic patients and HICs (Figure 3B). Indeed, HIV-specific CD8+ T cell responses were even barely detectable in some PTCs (Table S1).

We then examined the capacity of CD8+ T cells from the PTCs to suppress ex vivo the HIV-1 infection of autologous CD4+ T cells, as we recently showed that this test distinguishes the effective CD8+ T-cell responses found in many HICs from the ineffective responses in other patients [22]. The HIV-suppressive capacity of CD8+ T cells from the PTCs was poor (median decrease in p24: 0.39 log, Table S1), comparable with the capacity of cells from viremic patients (0.55 [0.43–1.00], p = 0.28) and treated patients (0.28 [0.12–0.86], p = 0.88) and far weaker than that observed in the HICs (1.63 [0.62–3.22], p<0.001) (Figure 3C). Of note, the capacity of CD8+ T cells from the PTCs to suppress HIV-1 infection was still weaker than that of the subset of HICs that did not bear the HLA-B*27 or B*57 alleles (1.55 [0.71–3.28], p = 0.002, n = 29).

### PTCs have a low T cell activation status

We then examined the activation status of CD4+ and CD8+ T cells from the PTCs by evaluating the expression of HLA-DR and CD38. Because of the low or undetectable frequency of HIV-specific cells that were detected using tetramers in these individuals, the analyses were limited to the total cell population (Figures 3D and S1). HLA-DR and CD38 expression, both separately and in combination, were very weak in the PTCs during the period of viral control without therapy and similar to that observed in patients on cART, as expected within the context of very low viremia [24]. These results contrasted with the strong HLA-DR expression observed on CD8+ T cells from the spontaneous HIV controllers (Figure 3D) [22], which has also been reported by others [25].

### PTCs have very low HIV reservoir levels that, in some cases, continue to decline for years after treatment interruption

**Figure 4:**
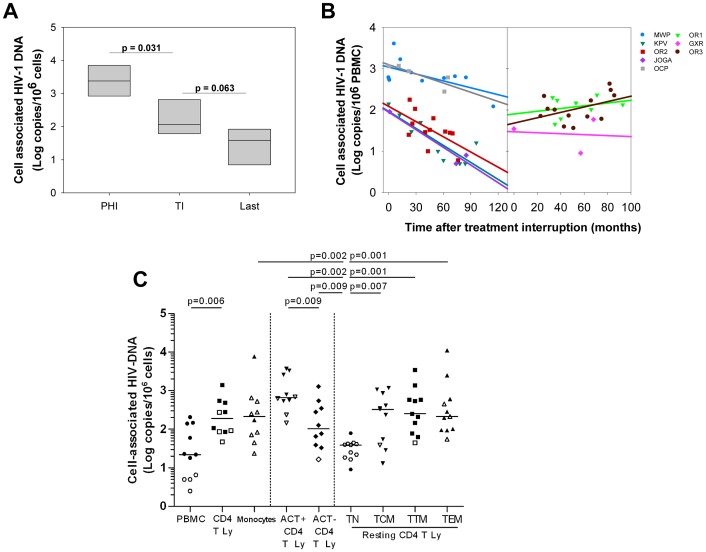
Post-treatment controllers have very low levels of cell-associated HIV DNA which keep decreasing after treatment interruption for some patients. **A.** Levels of cell-associated HIV-1 DNA (median and IQR) in 6 PTCs at PHI, just before or at treatment interruption (TI), and the last available value obtained at a median of 6 years after cART discontinuation (Last). **B.** The evolution of cell-associated HIV DNA after treatment interruption in PBMCs from 8 PTCs. The slope of the evolution of HIV-DNA levels after treatment interruption was calculated by linear regression (lines) of the available sequential measures (symbols). Five PTCs experienced a decline of their cell-associated HIV-DNA levels (left); two PTCs maintained stable levels and a positive slope was calculated for OR3 (right). **C.** Infection levels in various cell populations from 11 PTCs: PBMCs, CD4+ T cells and monocytes; activated and resting CD4+ T cells; resting naïve (TN), central memory (TCM), transitional memory (TTM) and effector memory (TEM) CD4+ T cell subsets (see Figure S2 for the sorting strategy). The open symbols represent values below the threshold of detection. The medians are represented. **A, B, C.** The results are expressed as the log10 HIV DNA copy numbers per million cells.

Overall, the PBMC-associated HIV-1 DNA levels in the PTCs during the infection control (median 1.71 log copies/10^6^ PBMC, Table 1) were similar to those in the HICs and much lower than those in patients with uncontrolled PHI or CHI or patients who started treatment during CHI [26], [27]. Sequential PBMC-associated HIV-1 DNA levels since PHI were available for 6 of the PTCs. In these PTCs, the HIV-1 DNA levels had declined strongly at or just before the treatment interruption (median 2,389 and 116 HIV-1 DNA copies/10^6^ cells at PHI and before treatment interruption, respectively; p = 0.031; Figure 4A). The last available value, at a median of 6 years after the cART interruption, tended to be even lower (39 HIV-1 DNA copies/10^6^ cells, p = 0.063; Figure 4A). Sequential PBMC-associated HIV-1 DNA levels were measured after treatment interruption for 8 PTCs (Figure 4B). The HIV DNA levels remained stable after cART discontinuation in two PTCs and a positive slope was observed for OR3, which is likely related to detectable viral replication at low levels in the last few years for this patient. In contrast, HIV DNA levels continued to progressively decline over the years in the five other PTCs in the absence of treatment. Thus, the PTCs had an extremely small viral blood reservoir, which in some cases continued to decline after long-term treatment interruption.

### PTCs have a low and inducible HIV reservoir distributed in the resting memory CD4 T cell subsets

We quantified the distribution of the HIV reservoir among various sorted lymphocyte populations of live peripheral cells available from 11 PTCs (Figure 4C). The HIV DNA was detectable in the PBMCs from 7 out of 11 PTCs and in total purified CD4+ T cells from 6 out of 10 PTCs (from whom enough cells were recovered). The results were either reported as the actual HIV DNA copy numbers/million cells or as an estimated value calculated as 50% of the detection threshold value when HIV DNA was not detected. As expected, the HIV DNA was 9-fold higher in the CD3+CD4+ T cells than in the total PBMCs (median 2.3 versus 1.3 log HIV DNA copies/million cells). Among the CD4+ T cells, activated CD25+69+HLA-DR+ CD4+ T cells were significantly more infected than resting CD4+ T cells (2.8 versus 2 log HIV DNA copies/million cells, p = 0.01). In contrast, the CD3−CD4+ monocytes were minimally infected, with the total cell-associated HIV DNA level detectable in only 2 out of 10 samples (estimated median 2.3 log HIV DNA copies/million monocytes). We also analyzed the reservoir distribution among the resting naïve (TN), central memory (TCM), transitional memory (TTM) and effector memory (TEM) CD4+ T cell subsets from 11 PTCs (Figure 4C). Cell-associated HIV DNA was detected in only 2 out of 11 samples in the resting naïve CD4 T cells (TN) (median 1.6 log HIV DNA copies/million TN, p = 0.001) and was lower than in resting memory CD4+ T cell subpopulations. In contrast, all resting memory CD4 T cells contained comparable levels of cell-associated HIV DNA (2.5, 2.4 and 2.3 median log HIV DNA copies/million in TCM, TTM and TEM cells, respectively).

**Figure 5:**
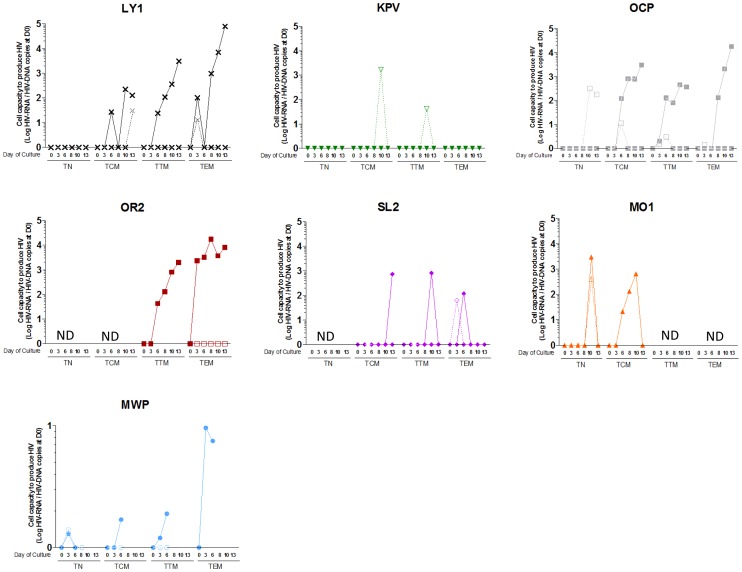
HIV replication is inducible from the resting memory CD4 T cell subsets from post-treatment controllers. The cell capacity to replicate HIV was evaluated in 7 PTCs (each symbol represents one PTC) by stimulating sorted-CD4 T cell subsets with an anti-CD3/anti-CD28 co-stimulation plus IL-2 and IL-7 (filled symbols and continuous lines) or IL-7 alone (empty symbols and dashed lines). HIV RNA was quantified in the supernatants of resting TN, TCM, TTM and TEM cells during a 13-day long culture. Results are expressed as the log10 of the ratio between the HIV RNA copy numbers quantified at a given day of culture and the level of cell-associated HIV DNA in the subset measured at D0 of culture. Kinetics of HIV production in a patient has been represented with connecting lines. HIV RNA values that were under the detection threshold of the technique were arbitrarily placed at 0. ND is not done.

To assess the presence of an inducible virus and the true nature of this HIV reservoir, we used anti-CD3 and anti-CD28 in the presence of IL-2 and IL-7 to stimulate the sorted resting CD4+ T cell subpopulations of 7 PTCs from whom an adequate number of cells was recovered (Figure 5). We observed a time-dependent virus production upon *in vitro* stimulation in 5 of the 6 sorted resting TCM, TTM and TEM subsets that were analyzed. We detected virus production from at least one T cell subset from each of the 7 tested patients. The failure to detect HIV production reflected the low number of HIV-infected cells added at baseline (a median of 6.5 HIV DNA copies in non-producing samples versus 97 HIV DNA copies when HIV RNA production could be detected). In line with the TN cells' extremely low infection levels, virus production in these cells was observed in only 2 out of 5 PTCs samples tested. The stimulation of a higher number of resting TN cells with IL-7 alone triggered virus production in 3 TN samples, despite undetected TN-associated HIV DNA in 2 cases (Figure 5).

### Long-lived resting CD4+ T cells contributed minimally to the HIV reservoir in the PTCs

**Figure 6:**
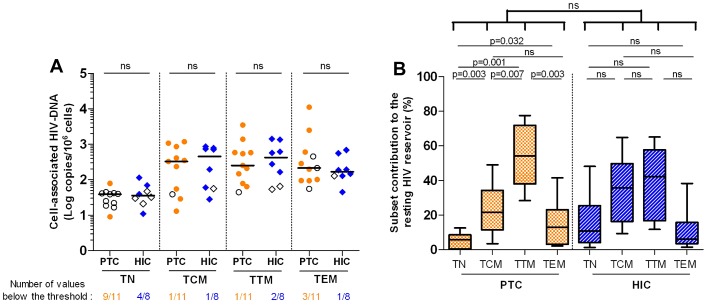
Weak contribution of long-lived resting CD4+ T cells to the HIV reservoir in the post-treatment controllers. **A.** HIV infection levels in the resting TN, TCM, TTM and TEM cells of 11 PTCs and 8 HICs. The results are expressed as the log10 HIV DNA copy numbers per million cells, and the medians are represented. The open symbols are values below the threshold of detection. ‘ns’ are non significant p values. **B.** CD4+ T cell subsets contribution to the resting HIV reservoir, considering both infection levels and frequency. The results are expressed as the median percentage of the resting CD4 HIV reservoir, with interquartile range [25%–75%] and minimum and maximum values. Statistical analyses were applied between all subsets from a single group as well as between each subset from the two groups.

We then compared the HIV reservoir distribution among the PTCs' resting CD4 T cell subsets to those of the HICs, whose total blood cells had similar low levels of HIV DNA. No differences were observed between the PTCs' resting CD4 T cell subsets' infection levels and those of the HICs (1.6, 2.7, 2.6 and 2.2 median log HIV DNA copies/million in the TN, TCM, TTM and TEM cells from HIC, respectively), except that the HIV DNA was undetectable in the TN cells from 9 out of 11 PTCs but only 4 out of 8 HICs (Figure 6A). To calculate each subset's contribution to the HIV reservoir, we evaluated the frequency of the resting CD4 T cell subsets in the blood (Figure S3). The predominance of the TTM subset in the PTCs drove the major contribution of this subset to the PTCs' resting CD4 T cell HIV reservoir (median 54%). This contribution of the TTM subset was significantly higher than that of the TCM which contributed to only 22%, the TEM (13%), and the TN subset which contributed very minimally to the resting HIV reservoir (6%; Figure 6B). In contrast, both TCM and TTM subsets contributed equally to the HIV reservoir in the HICs, as has been reported for other HIV-infected patients [28], [29]. Overall, such long-lived cells as the TN and TCM cells contributed very minimally to the PTCs' total HIV reservoir in resting CD4 T cells, which might have contributed to the gradual shrinking of the reservoir in some PTCs for whom the TTM subset was also the main contributor to the HIV reservoir (Figure S4).

### Higher-than-expected frequency of infection control after the interruption of long-term treatment initiated at PHI

**Figure 7:**
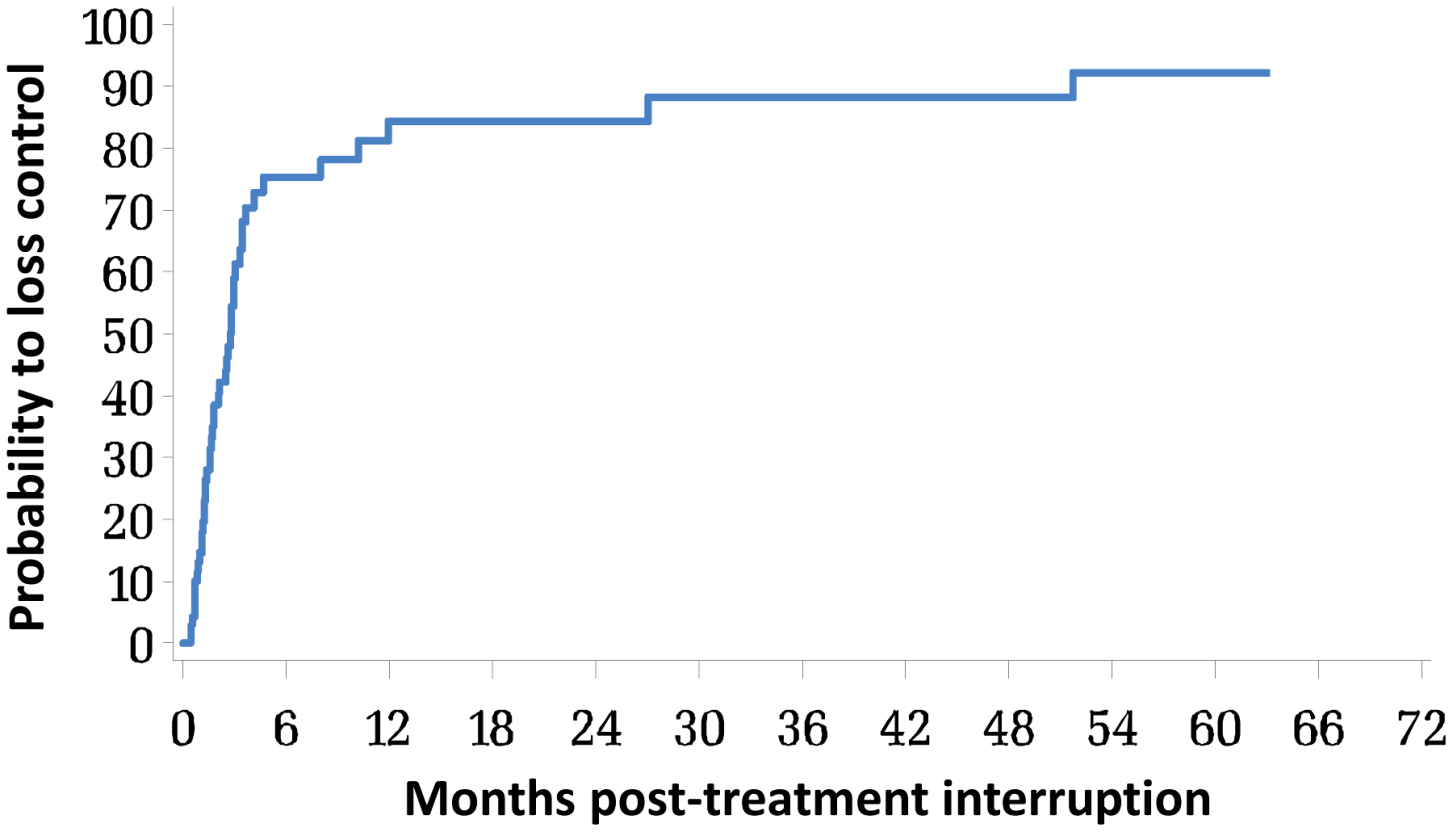
Interruption of long-term treatment initiated at PHI leads to a significant frequency of viremia control. Kaplan-Meier curve of the probability for patients included in the FHDH between 1997 and 2011 to lose control of viremia after interruption of a, at least, one year-long cART initiated within 6 months of HIV infection, and who had at least one viral load determination 12 months after treatment interruption (n = 74). Loss of control was defined by 2 or more viral loads above 50 RNA copies/mL or one viral load above 50 RNA copies/mL followed by resumption of cART.

PTCs may represent between 5 and 15% of patients with early cART interruption [15], [30], [31]. To better understand this phenomenon, we estimated its frequency of occurrence within the French Hospital Database on HIV (FHDH ANRS CO4) (http://www.ccde.fr/main.php?main_file=fl-1309272043-794.html). Between 1997 and 2011, 3,538 patients were included in the FHDH within 6 months of PHI. Among those, 1,013 patients were treated within 6 months post-infection, and 756 patients continued treatment for at least one year. Of those, only 70 patients with a viral load >50 copies/mL prior to treatment interrupted cART while their viral load was <50 copies/mL and with at least one viral load measurement recorded after treatment interruption. The mean number of viral load in the first three years post treatment interruption was 8 with a median delay of 3 months between 2 measurements. To estimate the probability of maintaining virological control, we used Kaplan-Meier estimates and defined loss of control as either 2 consecutive viral loads >50 copies/mL or 1 viral load >50 copies/ml, followed by cART resumption (Figure 7). The probability of maintaining viral control at 12 months was estimated as 15.3% [4.4–26.3], and it was identical at 24 months post-cART interruption.

## Discussion

Numerous efforts have been aimed to achieve a functional cure for HIV infection that would allow treatment to be stopped altogether. We studied 14 patients in whom viral replication was controlled to undetectable levels for several years after the discontinuation of cART. These PTCs with long-term virological remission may hold important clues about a possible functional cure for HIV.

The 14 PTCs presented in this study maintained lasting control of viremia after the interruption of prolonged therapy that began early during PHI. We found that most PTCs were readily distinguishable from spontaneous HICs in many respects. In many cases, spontaneous control seems to start very soon after HIV infection [16], [32], and most HICs have lower-than-normal viral loads during PHI [16]. In contrast, the PTCs had a more severe primary infection with higher viral loads and were frequently symptomatic, which may have prompted the early treatment in some cases. These observations are consistent with the generally unfavorable HLA genotypes of the PTCs. In particular, the risk alleles HLA B*35 and HLA-B*07, rarely observed in the HICs [17], were highly prevalent among the PTCs. Furthermore, two PTCs carried the HLA-B*3503 allele, which is associated with accelerated disease progression and impaired HIV-specific T cell function [33]. We cannot rule out the possibility that spontaneous control may have been masked in some cases by early therapy initiation. In particular, it might be possible that some potential HICs who lacked protective HLA alleles were more prone to have higher viral loads in primary infection and, hence, more likely to initiate therapy. However, other differences were observed between the PTCs and the HICs during the chronic phase of infection. In particular, the PTCs had a low frequency and quality of HIV-specific CD8+ T cell responses. Although some HICs do not exhibit strong HIV-specific CD8+ T cell responses [34], [35], the overall differences between the HICs and PTCs in our study were striking, even when the HICs carrying the protective HLA B*27 and B*57 alleles were excluded from the analyses. Finally, the PTCs were characterized by a lower CD8+ T cell activation status compared with the HICs.

The 5 to 15% of PTCs observed among the patients in the FHDH ANRS CO4 study and in other studies [15], [30], [31] appears higher than the proportion of HICs with spontaneous viral control in patients followed from primary infection [16], [36]. Therefore, our results strongly suggest that the infection control in the PTCs was not achieved spontaneously and was favored by the early onset of therapy. Because the interruption of long-term cART initiated early during PHI is not recommended, only a very small proportion (∼2%) of the patients in the FHDH experienced such an interruption, which may explain the rarity of PTCs worldwide. It is also important to consider that the 14 PTCs studied here had exhibited infection control without therapy for a very long period, and they may differ from PTCs with a shorter period of control [31].

The control of viremia following treatment interruption was associated with very low HIV blood reservoirs in the PTCs. This observation, together with similar observations in the HICs [26], suggests that limiting the pool of infected cells is crucial for the successful control of viral replication in the absence of therapy. In PTCs, the early cART initiation and the lengthy treatment period likely played an important role in reducing the reservoirs [7], [37]. Interestingly, five PTCs experienced a progressive decline in their viral reservoir after treatment interruption, which is one of the goals in the search for an HIV cure. However, very small HIV reservoirs do not guarantee infection control off therapy [38]. A key additional element might be a low reservoir distribution in cell subsets with long lifespan as naïve and central-memory T cells. Indeed we found that the cell subsets of all the PTCs analyzed ex vivo carried very low levels of HIV DNA. In particular, long-lived resting CD4+ T cells from the PTCs provided a minor contribution to the total HIV reservoir. Naïve CD4+ T cells were poorly infected, and overall the presence of the virus in these cells could not be detected (via DNA or viral replication) in 40% of the samples. This extremely low reservoir in PTCs' naïve cells contrasts with the massive infection detected at the end of the first month after initial infection with a median of 3 log copies HIV-DNA/million naïve cells (C. Bacchus and A. Cheret, personal communication), as also reported a year after initial infection in the absence of treatment, although the naïve cells contained a log lower level of cell-associated HIV DNA than other memory subsets [39]. These discrepancies suggest that early therapeutic intervention is extremely efficient at decreasing those very long-lived reservoirs.

Central memory CD4+ T cells also contributed very weakly to the HIV reservoir because of a skewed resting CD4+ T cell subset distribution with a large proportion of shorter-lived transitional memory cells. The skewed distribution of the resting CD4+ T cells observed in the PTCs is also found in uncontrolled early infection (our own unpublished results), further indicating that early therapeutic intervention strongly contributed to the nature of the viral reservoir in these individuals. The TCM cells have been shown to be heavily infected a year after infection, and the main contributor to the total HIV reservoir in patients treated during chronic infection [28]. Similarly weakly differentiated memory CD4 T cells were shown to contain the majority of the HIV reservoirs in untreated chronically infected patients [40]. In contrast, we recently reported a protection of TCMs that contributed less to the total HIV reservoir in long-term non progressors carrying HLA-B*27 or B*57 alleles [29], and TCM protection has also been observed in the nonpathogenic SIV infection of sooty mangabeys [41]. Altogether our results suggest that a functional cure would most likely require reducing both the size and the distribution of the HIV reservoirs, particularly among those resting CD4 T cells with a long lifespan or important clonogenic properties, such as naïve and central memory T cells.

Early therapy may also limit viral diversity and offer protection of innate and specific immunity from the deleterious effect of chronic immune activation. However, it remains unclear why only a limited fraction of patients is able to control the infection after therapy interruption, and a study of the effectors of control in PTCs is underway. In addition, mechanisms that diminish the susceptibility of host cells to HIV-1 infection [26] and protect long-lived cell types [42] have been implicated in the control of HIV/SIV infection and pathogenicity in humans and nonhuman primates and may favor infection control after treatment interruption in some individuals. Finally, it is also possible that properties of the viruses infecting the PTCs studied, along with potential limitation of viral diversity by early institution of cART may play a role in the phenotype reported. We are currently addressing these questions.

Arguments against cART initiation during PHI include the potential for long-term toxicity, the development of resistant viruses and the cost. However, new antiretroviral drugs are well tolerated, highly effective and associated with excellent compliance, strongly reducing the risk of resistance [43]. In addition, early treatment initiation improves survival [4] and reduces the risk of HIV-1 transmission [44]. Here, we show that in some HIV-infected individuals with symptomatic primary infection and no favorable genetic background, off-therapy viral control for several years may be associated with a very early and prolonged antiretroviral treatment. These findings argue in favor of early cART initiation and open up new therapeutic perspectives for HIV-1-infected patients.

## Methods

### Ethics statement

All of the subjects provided their informed written consent to participate in the study. The CO6 PRIMO, CO15 ALT and CO18 HIV controller cohorts are funded and sponsored by ANRS and were approved by the ethics review committees of Ile de France III, VI and VII, respectively. The institutional review board of Institut Pasteur and Pitié-Salpêtrière Hospital (Paris, France) also approved the study protocol. The VISCONTI study was funded by ANRS (EP47), sponsored by Orléans Regional Hospital and approved by the Tours ethics review committee.

### Subjects

The post-treatment controllers (PTCs) were defined as patients who initiated cART within 10 weeks of PHI and whose plasma HIV RNA levels remained less than 400 copies/mL for at least 24 months after cART interruption. Primary infection was defined as symptoms associated with an incomplete HIV-1 Western blot and a positive p24 antigen test or detectable plasma HIV RNA, and/or seroconversion documented by a positive HIV antibody test that was preceded by a negative test less than 3 months before. Fourteen PTCs were included in this study. Four had been identified in a previous study [15], six were recruited from the ANRS CO6 PRIMO cohort of patients diagnosed during PHI [31], and four were recruited from patient follow-up at Hôpital de la Croix Rousse in Lyon, CHRU Gui de Chauliac in Montpellier, and CHU de Saint Louis in Paris, France.

The HIV controllers (HICs) were patients from the ANRS CO15 and CO18 cohorts who had been infected for more than 5 years, were naïve of antiretroviral treatment and whose last 4 consecutive plasma HIV RNA values were less than 400 copies/ml. Viremic (VIR) patients were defined as patients who were HIV-1-infected for more than 6 months, were not receiving antiretroviral therapy and had HIV-1 plasma viral loads greater than 7500 RNA copies/ml. cART-treated individuals (ARTs) were HIV-1-infected patients whose viral load had been less than 50 RNA copies/ml of plasma for at least 6 months on cART initiated either on PHI or CHI.

### HLA typing

The subjects were serologically HLA-typed using complement-mediated lymphocytotoxicity testing (InGen One Lambda, Chilly Mazarin). High-definition genotyping of the HLA-B*35 alleles was conducted by direct exon sequencing.

### HIV-specific CD8+ T cell response

Interferon (IFN)-γ secretion by HIV-specific CD8+ T cells was quantified ex vivo with an ELISPOT assay [22]. For each subject, the optimal peptides (2 µg/mL) corresponding to known optimal CTL epitopes derived from the HIV-1 Env, Gag, Pol and Nef proteins were tested, depending on the results of the HLA typing.

The method used to assess the CD8+ T cells' capacity to suppress an ex vivo HIV-1 infection of autologous CD4+ T cells has been thoroughly previously described [45].

### Activation phenotyping

The following antibodies were used: CD8-APC-H7 or -PerCPCy5.5 (SK1), CD3-APC or -APC-H7 (SK7), HLA-DR-PE-Cy7 (L243) and CD38-PerCPCy5.5 (HIT2) (BD Biosciences). The cells were fixed and analyzed with a FACSCanto I flow cytometer (BD Bioscience).

### Sorting of the PBMC subpopulations

PBMCs that were cryopreserved and stored in liquid nitrogen and had more than 80% viability after thawing were sorted as live monocytes (CD3−CD4+) and activated and resting CD3+CD4+ T cells on a 5-laser FACS ARIA II cell sorter (Becton Dickinson) on the CyPS platform (UPMC), after staining with the following combination: Live-Dead Fixable Aqua (Life Technologies), CD3-Pacific Blue, CD4-AlexaFluor700, CCR7-PE Cyanine7 (3D12), CD27-APC, CD69-FITC and HLA DR-FITC (BD Pharmingen), CD45RA-ECD and CD25-FITC (Beckman Coulter). The resting CD4 T cells (CD25−CD69−HLADR−) were further sorted into the following categories: naïve (TN, CD45RA+CCR7+CD27+), central memory (TCM, CD45RA−CCR7+CD27+), transitional memory (TM, CD45RA−CCR7−CD27+), and effector memory (TEM, CD45RA−CCR7−CD27−) cells (Supplementary Figure S2). The collected cell numbers varied from 0.01 to 2 million cells among subsets and patients, and the purity of the sorted subsets was greater than 90%. The data were analyzed using Flowjo software (Treestar).

### HIV DNA quantification

The total cell-associated HIV DNA was quantified using ultrasensitive real-time PCR (Biocentric, Bantol, France) in the PBMC, monocyte and CD4 T cell subsets, as previously described (ANRS assay [46]). The entire HIV DNA extract was tested in two to four PCRs. The results are reported as either the actual HIV DNA copy numbers/million cells or as an estimated value calculated as 50% of the detection threshold value when the cell HIV DNA was lower than the threshold. The thresholds varied according to the available cell numbers and were calculated for each assay [47].

### Detection and amplification of HIV-1 from peripheral blood CD4+ T cell subsets

A first fraction of sorted resting CD4+ TN, TCM, TTM and TEM subsets from 7 PTCs was tested for the total cell-associated HIV DNA level (see above). A second fraction of the same samples was cultured in variable numbers in 10% FCS supplemented RPMI 1640 medium for 13 days after stimulation at Day 0 with anti-CD3/anti-CD28 plus IL-2 (Roche, 5 µg/ml) and human recombinant IL-7 (Cytheris, 1 ng/ml) or with human recombinant IL-7 alone. At Days 3, 6, 8, and 10, half of the supernatants were removed to quantify the HIV RNA, and IL-2 and IL-7 were added. The viral production kinetic in the supernatants was measured using real-time PCR HIV RNA quantification (Biocentric, Bandol, France). The viral production capacity of each subset was expressed as the ratio between the HIV RNA copies in the supernatants at a given day of culture and the level of cell-associated HIV DNA of each subset measured at Day 0 of culture.

### Statistical analyses

The Kruskal-Wallis nonparametric test was used to compare continuous variables between groups. A Wilcoxon matched-pairs signed rank sum test was used to compare variations in values (CD4+ T cell counts, HIV DNA levels) over time or to compare cell subsets in the sorting experiments. The allele frequencies in the different groups of patients were compared using Fisher's exact test. A Kaplan-Meier estimate was used to assess the probability of post-treatment control in patients who discontinued early cART. All values given in the text are medians and (range) or [IQR]. The SigmaStat 3.5 software (Systat Software Inc.-SSI, CA) or SAS software package, Version 9.2 (SAS Institute, Cary, NC, USA) was used for all analyses.

## Supporting Information

Figure S1 Similar CD4+ T cell activation levels in post-treatment controllers and HIV controllers. A. Percentage of CD4+ T cells from ART (n = 5), HIC (n = 58) and PTC (n = 8) that expressed CD38, HLA-DR or both CD38 and HLA-DR ex vivo. Means and standard deviations are shown. B. Surface CD38 and HLA-DR expression on CD4+ and CD8+ T cells from one representative ART, HIC and PTC.
(PDF)

Figure S2 Cell sorting scheme. Resting CD4 T cell subsets were selected out of a singlet lymphocyte population as assessed by cell size and structure. Live (Aqua-) resting CD3+CD4+ T cells (CD25−CD69−HLADR−) were further sorted as Naïve (TN, CD45RA+CCR7+CD27+), Central-Memory (TCM, CD45RA−CCR7+CD27+), Transitional-Memory (TM, CD45RA−CCR7−CD27+), and Effector-Memory (TEM, CD45RA−CCR7−CD27−) cells.
(PDF)

Figure S3 Different frequency of resting CD4+ T cell subsets in post-treatment controllers and HIV controllers. The frequency of TN, TCM, TTM and TEM cells among circulating resting CD4+ T cells in the PTCs and HICs.
(PDF)

Figure S4 Weak contribution of long-lived resting CD4+ T cells to the HIV reservoir in the post-treatment controllers with declining levels of cell associated HIV-DNA. CD4+ T cell subset contribution to the resting HIV reservoir for 4 PTC for whom we observed a diminution overtime on their HIV blood reservoir levels and HIC, taking into consideration both the cell infection levels and their frequency. Results are expressed as the median percentage of the resting CD4 HIV reservoir with interquartile range [25%–75%] and minimum and maximum values.
(PDF)

Table S1 HLA-class I alleles and characteristics of the CD8+ T cell response in the post-treatment controllers.
(PDF)

Table S2 Comparisons of relevant HLA allele frequencies in post-treatment controllers, HIV controllers and the reference French population.
(PDF)

Text S1 List of scientists and clinicians who are associated to the VISCONTI study.
(PDF)
