# Supplementary figures and images for: Post-Treatment HIV-1 Controllers with a Long-Term Virological Remission after the Interruption of Early Initiated Antiretroviral Therapy ANRS VISCONTI Study
Source: PLoS Pathog. 2013 Mar 14;9(3):e1003211. doi: 10.1371/journal.ppat.1003211 (PMC3597518; doi:10.1371/journal.ppat.1003211)

Figure S1

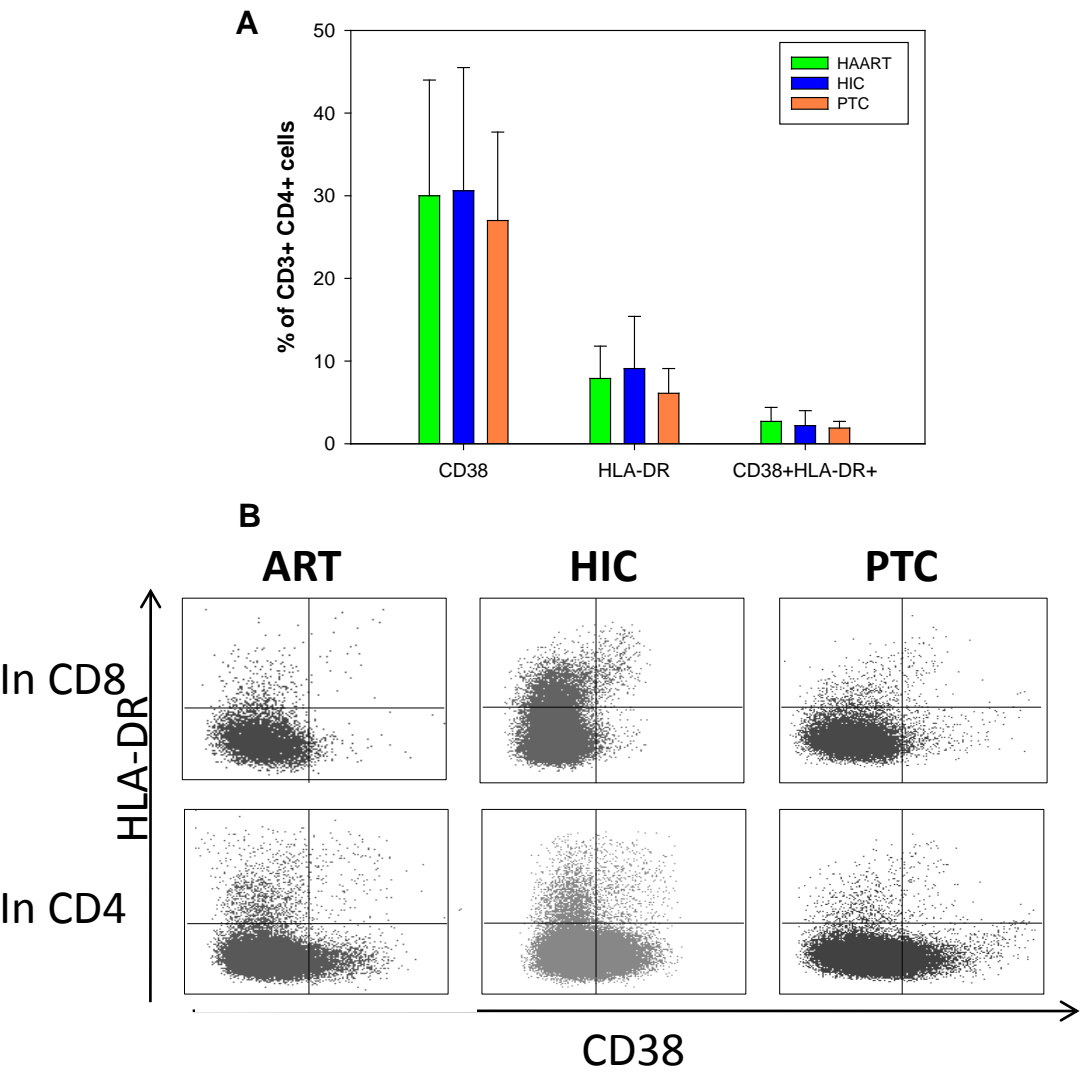

Supplement: Figure S1 — Similar CD4+ T cell activation levels in post-treatment controllers and HIV controllers. A. Percentage of CD4+ T cells from ART (n = 5), HIC (n = 58) and PTC (n = 8) that expressed CD38, HLA-DR or both CD38 and HLA-DR ex vivo. Means and standard deviations are shown. B. Surface CD38 and HLA-DR expression on CD4+ and CD8+ T cells from one representative ART, HIC and PTC. (PDF) [file ppat.1003211.s001.pdf]

Figure S2

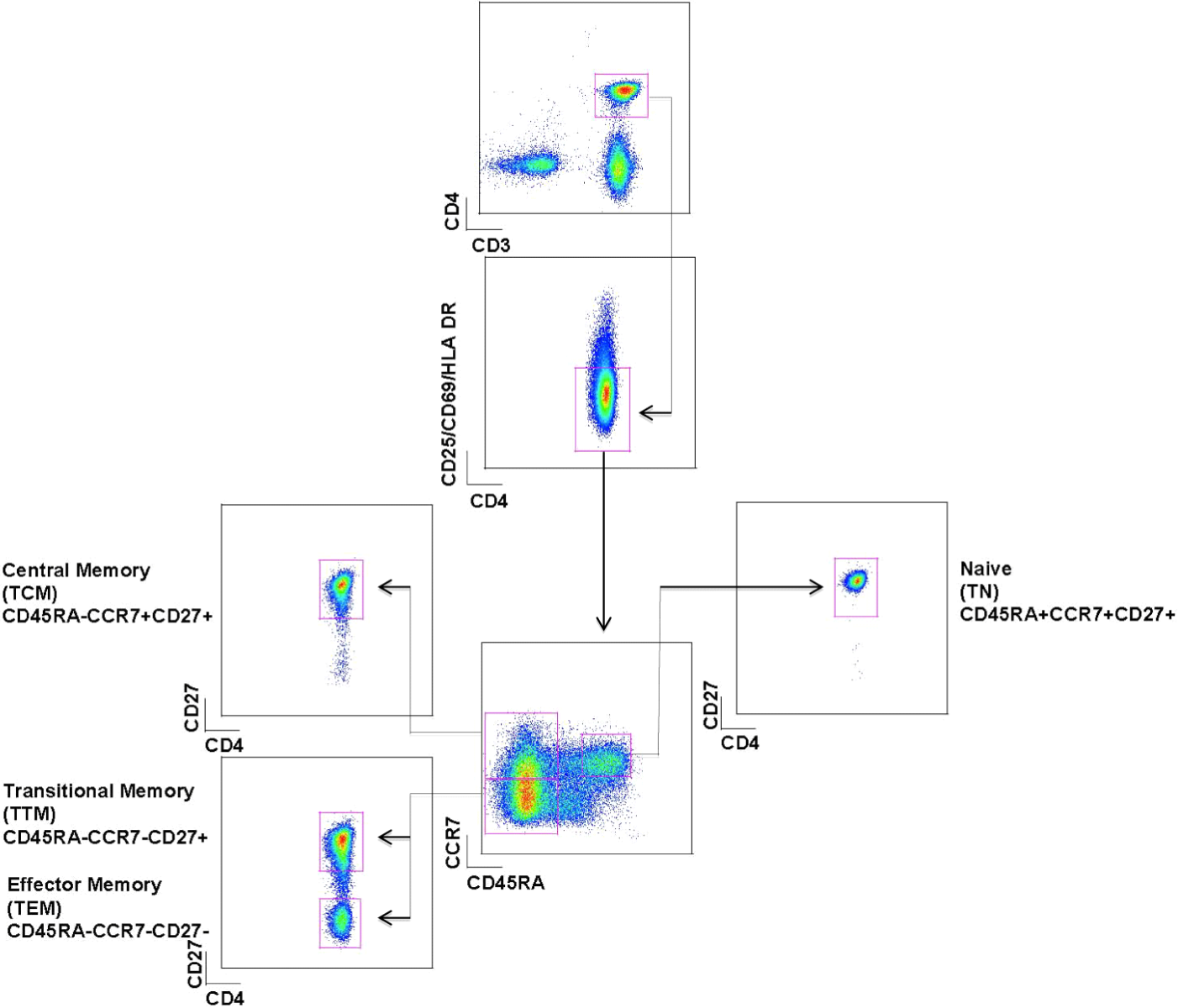

Supplement: Figure S2 — Cell sorting scheme. Resting CD4 T cell subsets were selected out of a singlet lymphocyte population as assessed by cell size and structure. Live (Aqua-) resting CD3+CD4+ T cells (CD25−CD69−HLADR−) were further sorted as Naïve (TN, CD45RA+CCR7+CD27+), Central-Memory (TCM, CD45RA−CCR7+CD27+), Transitional-Memory (TM, CD45RA−CCR7−CD27+), and Effector-Memory (TEM, CD45RA−CCR7−CD27−) cells. (PDF) [file ppat.1003211.s002.pdf]

Figure S3

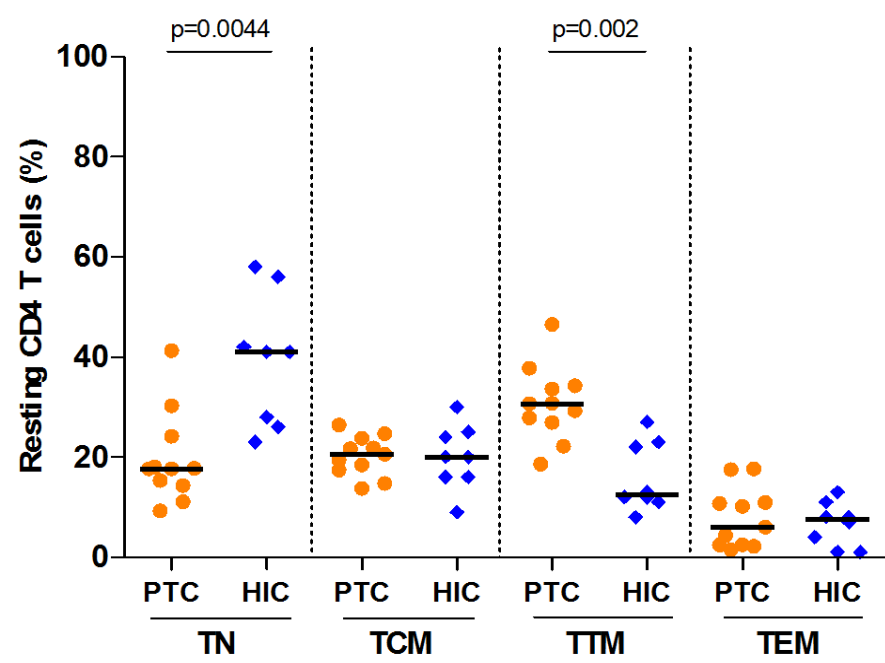

Supplement: Figure S3 — Different frequency of resting CD4+ T cell subsets in post-treatment controllers and HIV controllers. The frequency of TN, TCM, TTM and TEM cells among circulating resting CD4+ T cells in the PTCs and HICs. (PDF) [file ppat.1003211.s003.pdf]

Figure S4

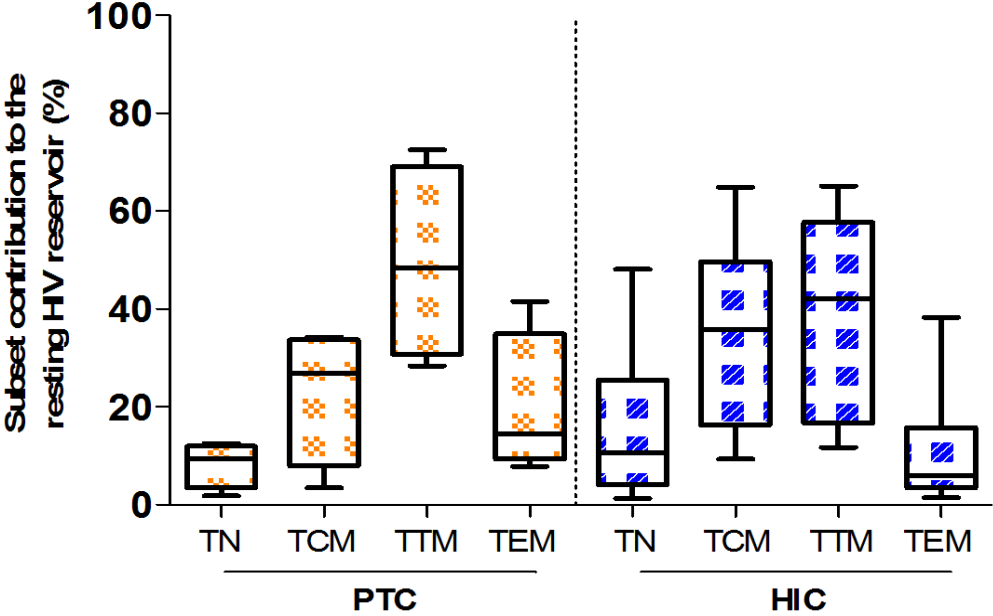

Supplement: Figure S4 — Weak contribution of long-lived resting CD4+ T cells to the HIV reservoir in the post-treatment controllers with declining levels of cell associated HIV-DNA. CD4+ T cell subset contribution to the resting HIV reservoir for 4 PTC for whom we observed a diminution overtime on their HIV blood reservoir levels and HIC, taking into consideration both the cell infection levels and their frequency. Results are expressed as the median percentage of the resting CD4 HIV reservoir with interquartile range [25%–75%] and minimum and maximum values. (PDF) [file ppat.1003211.s004.pdf]
