# Supplementary material for: Post-Treatment HIV-1 Controllers with a Long-Term Virological Remission after the Interruption of Early Initiated Antiretroviral Therapy ANRS VISCONTI Study
Source: PLoS Pathog. 2013 Mar 14;9(3):e1003211. doi: 10.1371/journal.ppat.1003211 (PMC3597518; doi:10.1371/journal.ppat.1003211)
Supplement: Table S1 — HLA-class I alleles and characteristics of the CD8+ T cell response in the post-treatment controllers. (PDF) [file ppat.1003211.s005.pdf]

Table S1. Characteristics of the CD8+ T cell response in PTC

Patient Code	HLA		log p24 decrease	ELISPOT (SFC/10^6 PBMC)
**OR1**	A*01/02	B*35/49	0.52	56
**OR2**	A*23/-	B*35/45	0.21	28
**OR3**	A*02/32	B*14/49	2.56	2065
**OR8**	A*29/30	B*44/47	1.18	ND
**KPV**	A*24/30	B*13/35	1.24	91
**GXR**	A*02	B*45/51	0.17	425
**CXK**	A*02/11	B*27/52	0.65	ND
**MWP**	A*03/24	B*35/44	0.00	420
**JOGA**	A*11/24	B*07/55	0.27	203
**OCP**	A*11/30	B*13/35	0.21	215
**LY1**	A*01/24	B*07/57	0.61	2345
**LY2**	A*11/24	B*07/27	0.22	260
**MO1**	A*02/03	B*18/51	2.87	553
**SL2**	A*01/26	B*05/13	0.04	0

ND: not done
