# Supplementary material for: Post-Treatment HIV-1 Controllers with a Long-Term Virological Remission after the Interruption of Early Initiated Antiretroviral Therapy ANRS VISCONTI Study
Source: PLoS Pathog. 2013 Mar 14;9(3):e1003211. doi: 10.1371/journal.ppat.1003211 (PMC3597518; doi:10.1371/journal.ppat.1003211)
Supplement: Table S2 — Comparisons of relevant HLA allele frequencies in post-treatment controllers, HIV controllers and the reference French population. (PDF) [file ppat.1003211.s006.pdf]

Table S2. HLA allele frequencies in PTC, HIC and the French population

**Alleles**	**French population frequency** N=6094[1]	**HIC frequency** N=148	**PTC frequency** N=28	***p* value**	**Pop vs HIC** **(*p* value)**	**Pop vs PTC** **(*p* value)**	**HIC vs PTC** **(*p* value)**
**B*27**	0.038	0.088	0.071	**0.006**	**0.004**	0.671	0.933
**B*57**	0.025	0.23	0.036	**<0.001**	**0.001**	0.808	**0.036**
**B*07**	0.115	0.047	0.107	**0.037**	**0.015**	0.868	0.418
**B*35**	0.089	0.047	0.179	**0.051**	0.105	0.185	**0.034**
**B*27+B*57**	0.063	0.318	0.107	**<0.001**	**0.001**	0.570	**0.042**
**B*07+B*35**	0.192	0.094	0.286	**0.003**	**0.001**	0.404	**0.013**

HIC : HIV controllers, PTC : Post-treatment controllers.

[1]from www.allelefrequecies.com ; [2in] bold statistically significant differences.
