# Supplementary material for: Post-Treatment HIV-1 Controllers with a Long-Term Virological Remission after the Interruption of Early Initiated Antiretroviral Therapy ANRS VISCONTI Study
Source: PLoS Pathog. 2013 Mar 14;9(3):e1003211. doi: 10.1371/journal.ppat.1003211 (PMC3597518; doi:10.1371/journal.ppat.1003211)
Supplement: Text S1 — List of scientists and clinicians who are associated to the VISCONTI study. (PDF) [file ppat.1003211.s007.pdf]

APPENDIX

The VISCONTI study group is composed by:

Brigitte Autran (UPMC, INSERM UMR-S 945, Paris), Veronique Avettand-Fenoel (AP-HP, CHU Necker-Enfants Malades, Paris), Charline Bacchus (UPMC, INSERM UMR-S 945, Paris), Frédéric Bastides (Hôpital Bretonneau, Tours), Loïc Bodard (IMM, Paris), Faroudy Boufassa (INSERM U1018, Le Kremlin-Bicetre), Corinne Brochier (Hopital Croix Rousse, Lyon), Bernard Cardon (Hôpital Tenon, Paris), Dominique Costagliola (UPMC, INSERM UMR_S 943, Paris), Laurent Cotte (Hôpital Croix Rousse, Lyon), Agnès Cros (Hotel Dieu, Paris), Yasmine Debab (CHU Rouen, Rouen), Barbara de Dieuleveult (CHR d'Orléans, Orléans), Jeannine Delgado (CHU St Louis, Paris), Christiane Deveau (INSERM U1018, Le Kremlin-Bicetre), Jacques Durant (CHU de Nice, Nice), Isabelle Girault (INSERM U1022, Le Kremlin-Bicetre), Cecile Goujard (AP-HP, Hôpital de Bicêtre, Le Kremlin Bicêtre), Julien Guergnon (UPMC, INSERM UMR-S 945, Paris), Laurent Hocqueloux (CHR Orleans, Orleans), Corinne Jadand (Hôpital Bichat, Paris), Caroline Lascoux-Combes (CHU St Louis, Paris), Camille Lecuroux (INSERM U1022, Le Kremlin-Bicetre), Aline Maignan (Hotel Dieu, Paris), Adeline Melard (AP-HP, CHU Necker-Enfants Malades, Paris), Corinne Merle de Boever (CHRU Gui de Chauliac, Montpellier), Laurence Meyer (INSERM U1018, Le Kremlin-Bicetre), Patrick Miailhes (Hôpital Croix Rousse, Lyon), Sophie Pailhes (Hopital Croix Rousse, Lyon), Gianfranco Pancino (Institut Pasteur, Paris), Thierry Prazuck (CHR Orleans, Orleans), Valerie Potard (UPMC, INSERM UMR_S 943, Paris), Christine Rouzioux (AP-HP, CHU Necker-Enfants Malades, Paris), Asier Sáez-Cirión (Institut Pasteur, Paris), Assia Samri (UPMC, INSERM UMR-S 945, Paris), Daniel Scott-Algara (Institut Pasteur, Paris), Martine Sinet (INSERM U1022, Le Kremlin-Bicetre), Ioannis Theodorou (UPMC, INSERM UMR-S 945, Paris), Alain Venet (INSERM U1022, Le Kremlin-Bicetre), Pierre Versmisse (Institut Pasteur, Paris), Jean-Paul Viard (Hotel Dieu, Paris), David Zucman (CHU Foch, Suresnes).
